# Lateral plate mesoderm cell-based organoid system for NK cell regeneration from human pluripotent stem cells

**DOI:** 10.1038/s41421-022-00467-2

**Published:** 2022-11-08

**Authors:** Dehao Huang, Jianhuan Li, Fangxiao Hu, Chengxiang Xia, Qitong Weng, Tongjie Wang, Huan Peng, Bingyan Wu, Hongling Wu, Jiapin Xiong, Yunqing Lin, Yao Wang, Qi Zhang, Xiaofei Liu, Lijuan Liu, Xiujuan Zheng, Yang Geng, Xin Du, Xiaofan Zhu, Lei Wang, Jie Hao, Jinyong Wang

**Affiliations:** 1 State Key Laboratory of Stem Cell and Reproductive Biology, Institute for Stem Cell and Regeneration, Institute of Zoology, Chinese Academy of Sciences, Beijing, China; 2 CAS Key Laboratory of Regenerative Biology, Guangzhou Institutes of Biomedicine and Health, Chinese Academy of Sciences, Guangzhou, Guangdong China; 3 University of Chinese Academy of Sciences, Beijing, China; 4 Beijing Institute for Stem Cell and Regenerative Medicine, Beijing, China; 5 Department of Hematology, Guangdong General Hospital, Guangzhou, Guangdong China; 6 Department of Pediatrics, State Key Laboratory of Experimental Hematology, National Clinical Research Center for Blood Diseases, Institute of Hematology & Blood Diseases Hospital, Chinese Academy of Medical Sciences & Peking Union Medical College, Tianjin, China; 7 National Stem Cell Resource Center, Chinese Academy of Sciences, Beijing, China

**Keywords:** Stem-cell differentiation, Tumour immunology

## Abstract

Human pluripotent stem cell (hPSC)-induced NK (iNK) cells are a source of off-the-shelf cell products for universal immune therapy. Conventional methods for iNK cell regeneration from hPSCs include embryoid body (EB) formation and feeder-based expansion steps, which are time-consuming and cause instability and high costs of manufacturing. Here, we develop an EB-free, organoid aggregate method for NK cell regeneration from hPSCs. In a short time-window of 27-day induction, millions of hPSC input can output over billions of iNK cells without the necessity of NK cell expansion feeders. The iNK cells highly express classical toxic granule proteins, apoptosis-inducing ligands, as well as abundant activating and inhibitory receptors. Functionally, the iNK cells eradicate human tumor cells via mechanisms of direct cytotoxicity, apoptosis, and antibody-dependent cellular cytotoxicity. This study provides a reliable scale-up method for regenerating human NK cells from hPSCs, which promotes the universal availability of NK cell products for immune therapy.

## Introduction

Immunotherapy provides a new paradigm for saving patient lives with malignancies. Adoptive transfer of natural T cells or NK cells already shows significant therapeutic effects in patients with several tumor types, including CD19^+^ B cell malignancies, breast cancer, and ovarian cancer^1,2^. However, immunotherapy still faces problematic issues related to limited cell source, economic costs, and urgent manufacturing necessity, as well as low efficiency of gene editing/engineering^3–5^. Induced NK (iNK) cells from human pluripotent stem cells (hPSCs), including embryonic stem cells (ESCs) and induced pluripotent stem cells (iPSCs), are ideal to overcome the above-mentioned problems^4,6^. Of note, human ESCs, as natural early-stage pluripotent stem cells, have safety advantages over iPSCs due to rare gene mutation accumulations and avoidance of reprogramming process by exogenous genes^7,8^.

Unlike T cells, NK cells have unique advantages in immune therapy due to little toxicities and no stringent requirement of HLA matches^3^. NK cells inherit the power to directly kill tumor cells that typically escape T-cell killing by downregulating HLA class I (MHC-I) molecules. In the presence of therapeutic monoclonal antibodies, NK cells can further improve their anti-tumor efficacy via antibody-dependent cellular cytotoxicity (ADCC) mechanism^9^. hPSC-derived NK cells even showed promising efficacy in eliminating tumor cells in vivo^10,11^. Hence, regenerative NK cells from hPSCs are considered as an ideal “off-the-shelf” immune cell product for translational medicine.

Encouragingly, hPSCs have been successfully induced into hematopoietic and immune lineage cells in vitro via an interim step of embryoid body (EB) formation^12–15^. Hematopoietic lineages originate from the lateral plate mesoderm (LPM) during embryonic development^16^. Conventionally, EB formation and monolayer differentiation methods can both generate mesoderm progenitors from hPSCs with low and variable efficiencies^17,18^. Thus, the elevation of the yields of LPM cells may improve the terminal yields of regenerative immune cells from hPSCs. The OP9 feeder cell line (M-CSF deficient) and derivatives are commonly used for hematopoietic differentiation and lymphogenesis in vitro^19^. Fetal thymic organ cultures (FTOCs) show great advantages over monolayer induction system in T cell induction in vitro^20,21^. Artificial thymic organoid (ATO) culture also promotes the T cell regeneration from hPSCs^22,23^. Thus, three-dimensional structure of stromal cells benefits hemogenesis and lymphogenesis.

In this study, we developed an EB-free, organoid aggregate method for NK cell regeneration from hPSCs. We firstly optimized the production efficiency of LPM intermediates, which are essential for the initiation of NK-specific organoid aggregates. Secondly, organoid aggregates were prepared by mixing LPM and OP9 stromal cells, and were induced toward NK cell following conventional NK cell induction. In a short time-window of 27-day induction, one million hPSCs can produce over one billion iNK cells without the necessity of NK cell expansion feeders. The iNK cells exhibited strong anti-tumor activity in animal models bearing human tumor cells.

## Results

### Efficient induction of LPM from hPSCs

**Fig. 1:**
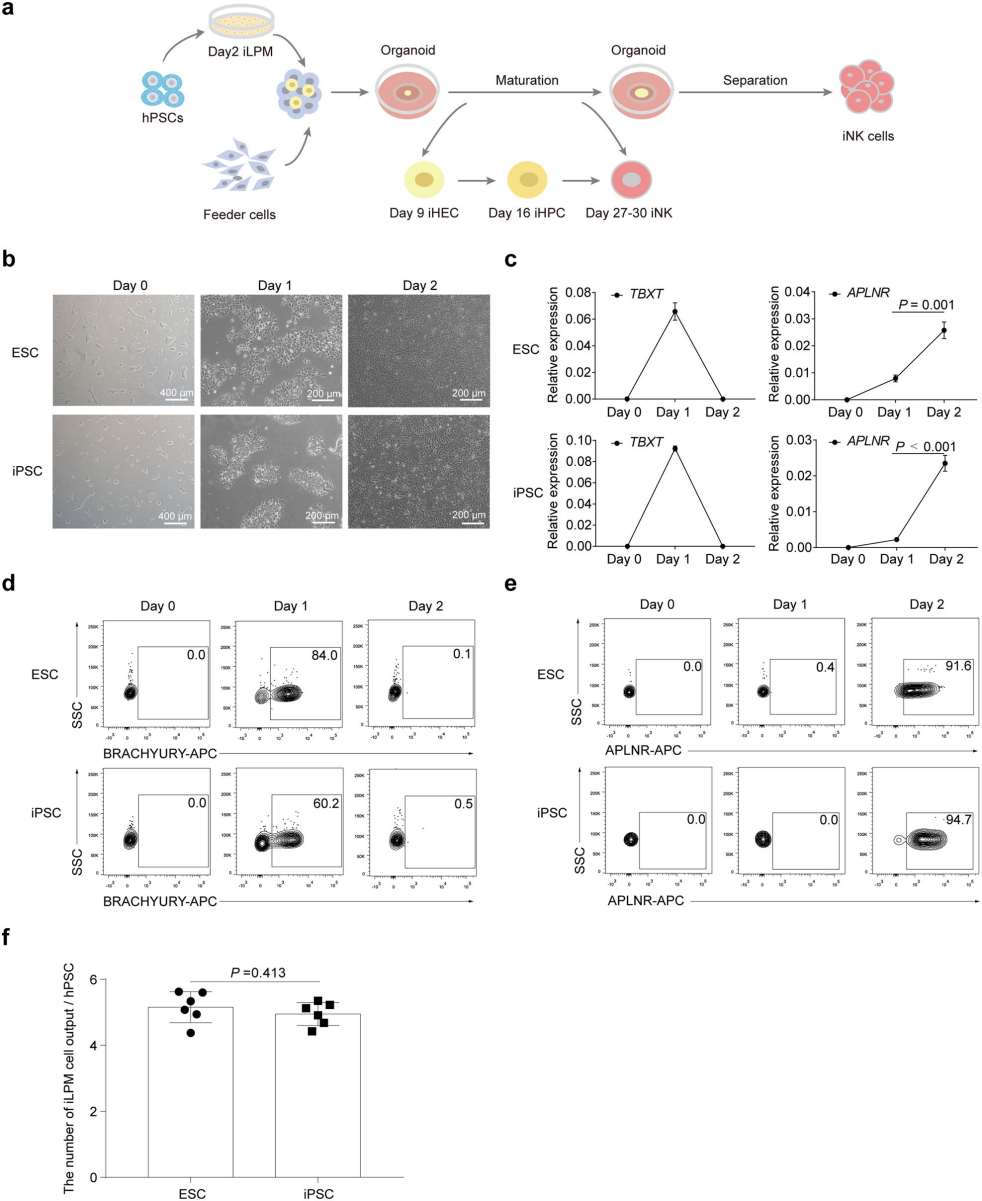
LPM induction from hPSCs. **a** Schematic diagram of NK cell regeneration. **b** Cell images at specified time points. **c** Real-time quantitative PCR analysis of *TBXT* and *APLNR* gene expression levels at selected time points. *β-ACTIN* gene was used as internal control. The relative gene expression levels were calculated as 2^–ΔCt^. **d** Dynamic analysis of BRACHYURY^+^ primitive streak (PS) cells at selected time points. **e** Dynamic analysis of the APLNR^+^ LPM cells. **f** Statistics of iLPM cell yields per hPSC input. *n* = 6 each group. Data were collected from two independent experiments. Two-tailed independent *t*-test. ESC: human ESC line hPSC-2; iPSC: human iPSC line hPSC-6.

A typical phenomenon in hematopoietic induction in petri dishes is that primitive hematopoietic progenitor cells (HPCs) lacking lymphoid potential are always predominant^24,25^. Definitive hematopoietic cell fate commitment in natural embryos undergoes sequential events of LPM formation, hemogenic endothelial cell (HEC) specification, endothelial to hematopoietic transition (EHT), and the emergence of definitive hematopoietic progenitors^16,26^. To address the issue of low efficiency of NK lymphogenesis, we combined optimization strategies and established a scheme for regenerating NK cells from hPSCs, which comprises stepwise generation of abundant early-stage LPM cells^27–29^, HECs, definitive HPCs^22^, and mature NK cells^30^ (Fig. 1a). Cell images and real-time quantitative PCR results showed that hPSCs began to migrate on the first day of differentiation in petri dishes, and highly expressed the primitive streak-specific gene *TBXT*^31^ (Fig. 1b, c). On the second day, the cells proliferated rapidly, and expressed LPM markers *APLNR*^32^ and *HAND1*^27^. The paraxial mesoderm-specific gene *MSGN1* was not activated during the entire LPM induction process^27^ (Fig. 1b, c; Supplementary Fig. S1a, b). Flow cytometry analysis also showed that > 60% of the cells on the first day of differentiation expressed the BRACHYURY protein, which is encoded by the *TBXT* gene. On the second day of differentiation, > 90% cells expressed the APLNR (Fig. 1d, e; Supplementary Fig. S1c, d). Moreover, one hPSC can output 5.1 ± 0.4 (mean ± SD) the induced lateral plate mesoderm (iLPM) cells on average (Fig. 1f). Thus, the iLPM cells are efficiently produced from hPSCs within 48-h monolayer induction.

### Organoid aggregate system for NK cell regeneration

**Fig. 2:**
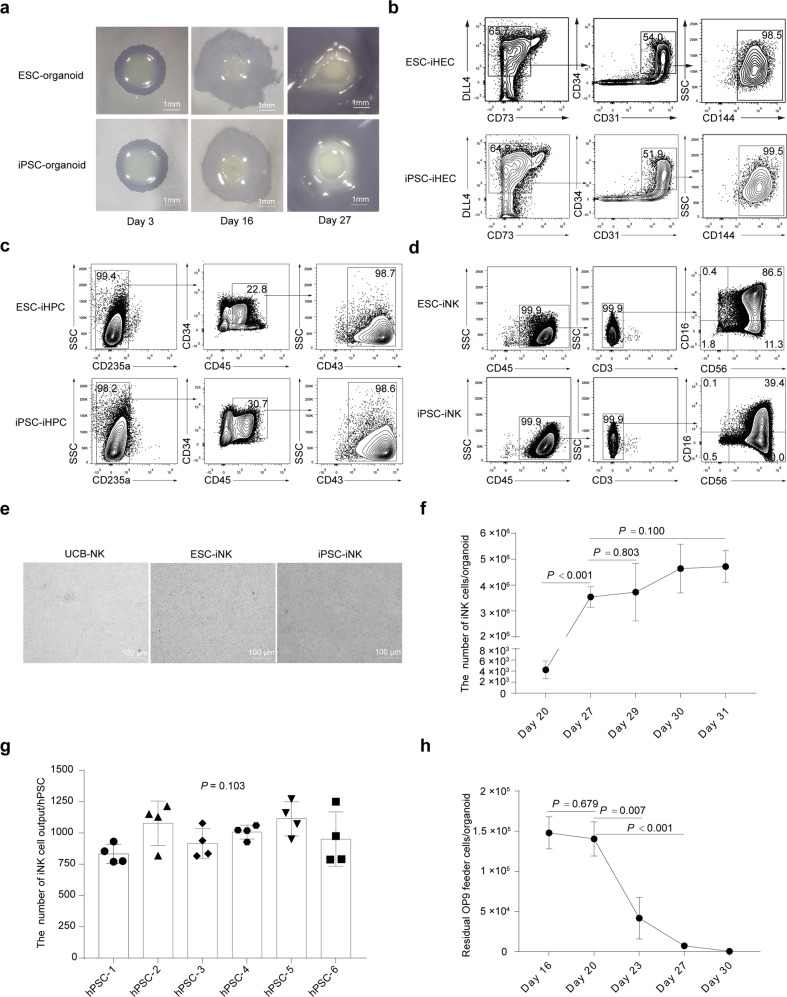
Stepwise induction of iNK cells from LPM cells using organoid system. **a** Organoid aggregate images at specified time points. **b** Immuno-phenotypes of hemogenic endothelial cells (iHECs, defined as CD73^–^DLL4^+^CD31^+^CD34^+^CD144^+^) on Day 9. **c** Immuno-phenotypes of hematopoietic progenitor cells (iHPCs, defined as CD235a^–^CD45^+^CD34^+^CD43^+^) on Day 16. **d** Immuno-phenotypes of iNK cells (CD45^+^CD3^–^CD56^+^CD16^+/–^) on Day 27. **e** The images of NK cells separated from organoid aggregates on Day 27. **f** The dynamic quantities of iNK cells (CD45^+^CD3^–^CD56^+^) at the indicated time points (*n* = 3 each group). **g** Statistics of yields of iNK cells (Day 27) induced from different hPSC lines. *n* = 4 each group. Data were collected from four batches of induction experiments using five human ESC lines (hPSC-1, hPSC-2, hPSC-3, hPSC-4, and hPSC-5) and one iPSC line (hPSC-6). One point represents the mean of iNK cell output from three repeats per batch induction experiment. **h** The residual OP9 feeder cells at the selected time points (*n* = 3 each group). Two-tailed independent *t*-test. Mann-Whitney *U* test (**f**), and one-way ANOVA (**g**).

We combined the iLPM cells and OP9 feeder cells to prepare organoid aggregates on Day 2. The images of organoid aggregates on Day 3, Day 16, and Day 27 were shown (Fig. 2a). Further, we observed the occurrence of abundant induced hemogenic endothelial cells (iHECs, CD144^+^ in CD73^–^DLL4^+^CD31^+^CD34^+^: > 93.6%) on Day 9, and induced hematopoietic progenitor cells (iHPCs, CD43^+^ in CD235a^–^CD45^+^CD34^+^: > 97.3%) on Day 16^33^. On Day 27, 97.4% ± 2.6% (mean ± SD) cells (CD45^+^CD3^–^CD56^+^CD16^+/–^) already showed the terminal mature NK cell phenotypes^34^, which were defined as induced NK (iNK) cells (Fig. 2b–d; Supplementary Fig. S2a–c). Morphologically, ESC-iNK cells and iPSC-iNK cells were similar to the activated umbilical cord blood NK (UCB-NK) cells (Fig. 2e). Statistically, the organoid aggregates produced 3.5 ± 0.4 (mean ± SD) million CD56^+^ iNK cells on Day 27 and reached 4.1 (median) million on Day 30 (Fig. 2f). We tested six hPSC lines derived from six individuals and all these stem cell lines reproducibly generate abundant iNK cells (*P* < 0.001) (Fig. 2g). On average, one hPSC can output 983.6 ± 32.4 (mean ± SD) iNK cells on Day 27 (Fig. 2g). Interestingly, the residual OP9 feeder cells decreased sharply on Day 20 and were barely detectable on Day 30 (*P* < 0.001) (Fig. 2h). In addition, we assessed multiple OP9-derived cell lines for organoid aggregates, including OP9-hDLL1 and OP9-hDLL4, which showed comparable iNK cell regeneration efficiencies (Supplementary Fig. S2d). We further performed side-by-side experiments using OP9 or OP9-hDLL4 as feeder cells and assessed the induction efficiency, maturation, and cytotoxicity of iNK cells from ESCs and iPSCs. Both OP9 and OP9-hDLL4 supported efficient maturation of iNK cells (CD45^+^CD3^–^CD56^+^) on Day 27 (ESC-iNK, 95.0% ± 2.1% (mean ± SD), *n* = 6, *P* = 0.528; iPSC-iNK, 95.2% ± 1.2% (mean ± SD), *n* = 6, *P* = 0.093) (Supplementary Fig. S3a–d). In addition, using OP9 and OP9-hDLL4 as feeder cells resulted in similar absolute yields of iNK cells (ESC-iNK, 3,759,255 ± 304,568 (mean ± SD), *n* = 6, *P* = 0.142；iPSC-iNK, 3,657,048 ± 396,339 (mean ± SD), *n* = 6, *P* = 0.744) (Supplementary Fig. S3e, f). We also simultaneously performed anti-tumor activity assay using iNK cells from OP9 and OP9-hDLL4 feeder groups starting from the same stem cell lines. Our data showed that iNK cells from OP9 and OP9-hDLL4 groups showed similar tumor-killing activity against ovarian tumor cell line A1847 and leukemia cell line K562 (Supplementary Fig. S3g–j). Collectively, an organoid aggregate system effectively supports NK cell regeneration starting from LPM cells.

### iNK cells express specific activating receptors, inhibitory receptors, and effector molecules

**Fig. 3:**
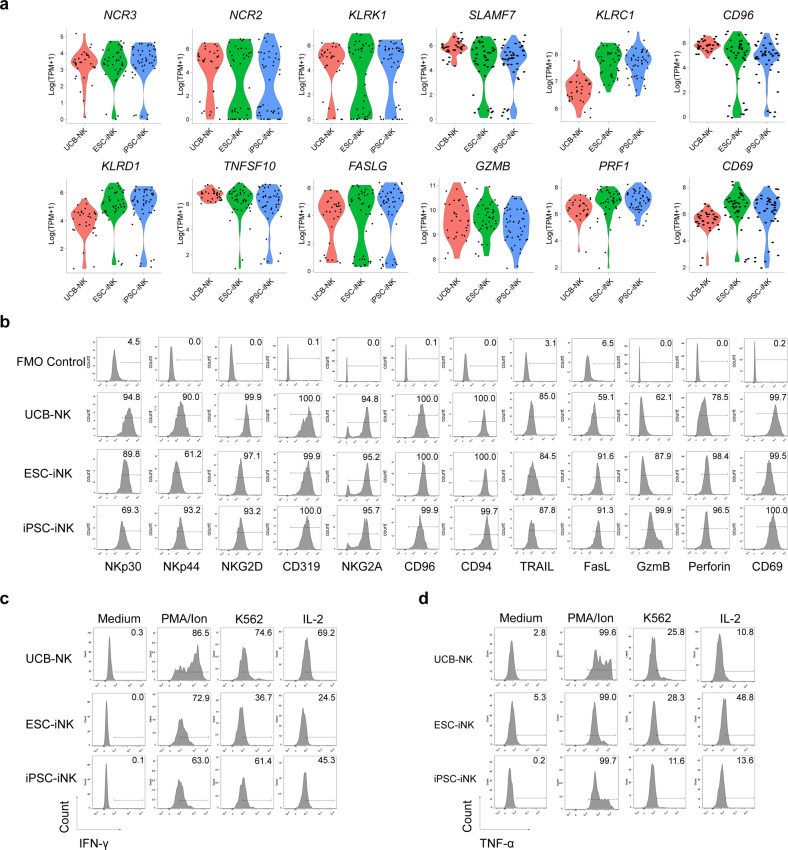
Molecular features of ESC-iNK, iPSC-iNK, and UCB-NK cells. **a** Violin plots show the expression profiles of the indicated NK cell surface receptors and effectors (*NCR3*, *NCR2*, *KLRK1*, *SLAMF7*, *KLRC1*, *CD96*, *KLRD1*, *TNFSF10*, *FASLG*, *GZMB*, *PRF1*, and *CD69*) in ESC-iNK cells (*n* = 41), iPSC-iNK cells (*n* = 47), and UCB-NK cells (*n* = 32). The expression value (TPM) of each gene was transformed with log_2_. One point represents one cell. **b** The expression levels of NK cell typical receptors and effectors (NKp30, NKp44, NKG2D, CD319, NKG2A, CD96, CD94, TARIL, FasL, GzmB, Perforin, and CD69) were analyzed by flow cytometry. **c**, **d** IFN-γ (**c**) and TNF-α (**d**) production by iNK cells upon stimulation with PMA/ionomycin, K562 tumor cells, or recombinant human IL-2. IFN-γ^+^ or TNF-α^+^ cells were gated on CD45^+^CD3^–^CD56^+^ NK cells. ESC: human ESC line hPSC-2; iPSC: human iPSC line hPSC-6.

To illustrate the molecular features of iNK cells, we performed single-cell RNA sequencing (scRNA-seq) of iNK cells and natural control NK cells (UCB-NK cells). The expression patterns of activating receptors and inhibitory receptors determine the activating status and functionalities of NK cells^35,36^. As expected, both ESC-iNK cells and iPSC-iNK cells expressed classical activating and inhibitory receptor genes, including activating receptor genes *NCR3*, *NCR2*, *KLRK1*, and *SLAMF7*, and inhibitory receptor genes *KLRC1* and *CD96*^36,37^ (Fig. 3a). We also confirmed the expression of these receptors on iNK cells at protein levels by flow cytometry analysis. The type II transmembrane protein CD94 dimerizes with NKG2A to form CD94/NKG2A heterodimer, which recognizes non-classical HLA-E class I molecules and inhibits signal activation, or combines with NKG2C to form CD94/NKG2C receptor to transmit activating signals and promote NK cell activation^38^. Indeed, the CD94 molecules on the surface of iNK cells were abundant (CD94^+^ in CD56^+^: > 99.7%) (Fig. 3b). In addition, the iNK cells also highly expressed the essential effector molecules, including apoptosis-related ligands (TRAIL and FasL), cytotoxic granules (GzmB and perforin), and activating molecule CD69 (Fig. 3a, b). Moreover, we performed 10× scRNA-seq analysis of NK cells induced from ESCs and iPSCs and carried out transcriptome comparison with naturally resting NK cells and activated NK cells from human peripheral blood. The data demonstrated that the ESC- and iPSC-derived iNK cells projected well to the activated NK cells from human peripheral blood (Supplementary Fig. S4a). Similar to naturally activated NK cells, there is no obvious heterogeneity among iNK cells derived from ESC and iPSC cells (Supplementary Fig. S4b). However, heterogeneous NK cell populations were indeed observed among resting NK cells from human peripheral blood, which differed from the activated NK cells either from ESCs, iPSCs, or activated NK cells from human peripheral blood (Supplementary Fig. S4b). Furthermore, the iNK cells can produce IFN-γ and TNF-α in the presence of PMA/ionomycin, K562 tumor cells, or IL-2 (Fig. 3c, d). Collectively, iNK cells express typical NK cell markers and effector molecules.

### iNK cells eliminate tumor cells in vitro and possess ADCC activity

**Fig. 4:**
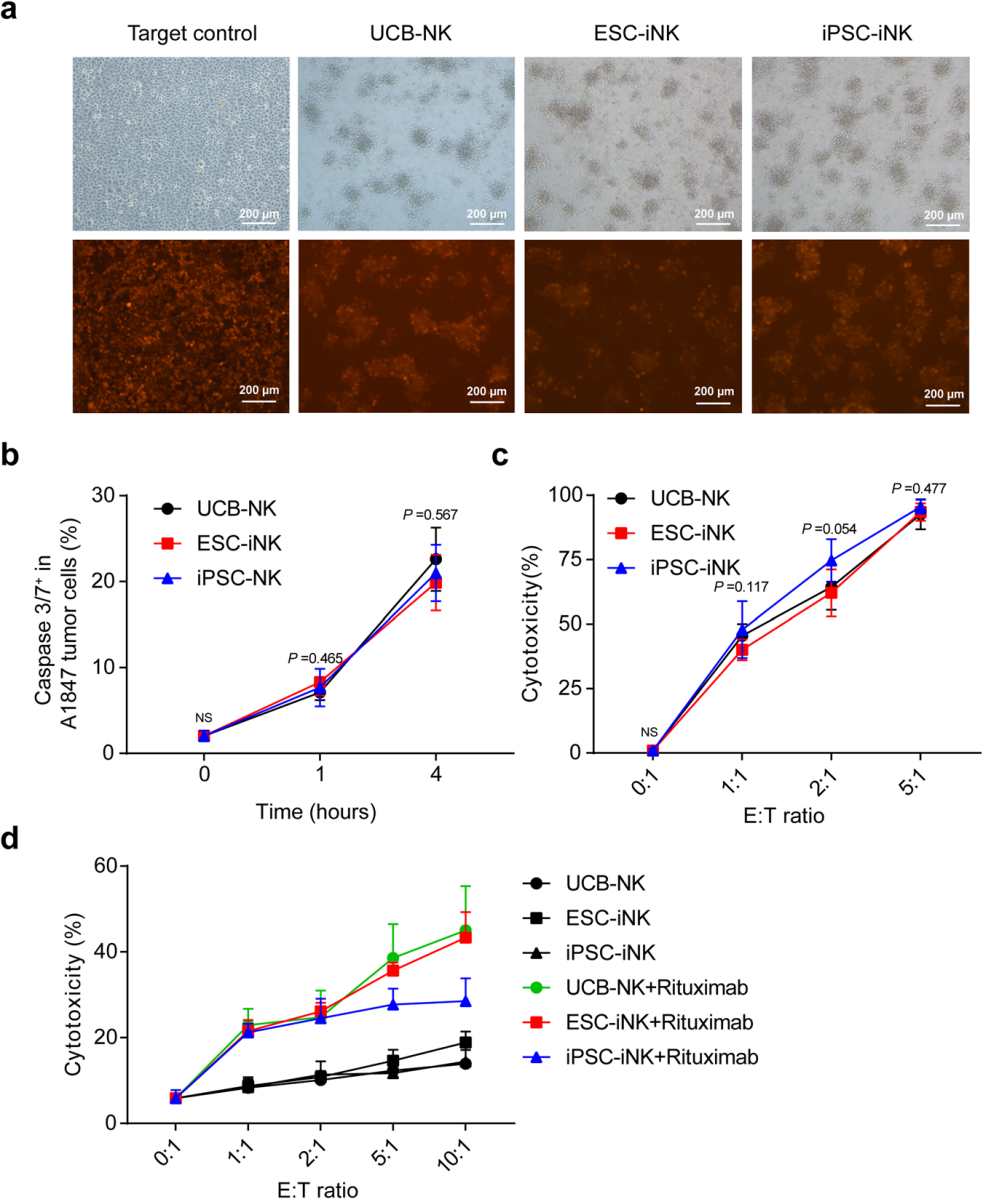
iNK cells exhibit tumor-killing activities in vitro. **a** Representative images of residual A1847-tdTomato^+^ tumor cells (Target, T) after 4-h co-culture with iNK cells (Effector, E) (E:T = 5:1). **b** Flow cytometry analysis of apoptotic A1847-tdTomato^+^ tumor cells post co-culture with iNK cells (E:T = 2:1) for 1 h and 4 h. *n* = 6 each group. **c** Cytotoxicity analysis of the iNK cells. iNK cells were co-cultured with CFSE-labeled A1847 tumor cells for 4 h at the indicated effector to target ratios. *n* = 6 each group. **d** ADCC analysis of iNK cells. iNK cells were co-cultured with CFSE-labeled Raji cells in the presence of anti-CD20 antibody (Rituximab, 20 μg/mL) for 4 h at the indicated effector to target ratios. *n* = 9 each group. Data were collected from two (**b**, **c**) or three (**d**) independent experiments. One-way ANOVA and Kruskal-Wallis tests were used for statistics. NS, not significant. ESC: human ESC line hPSC-2; iPSC: human iPSC line hPSC-6.

Unlike adaptive T cells, NK cells can directly recognize and kill tumor cells with downregulated HLA class I (MHC-I) expression^39^. Therefore, we chose an NK cell-sensitive human ovarian cancer cell line A1847 (tdTomato^+^) for validation of the tumor-killing ability of iNK cells. ESC-iNK cells, iPSC-iNK cells, and UCB-NK cells at sequential doses (Effectors (E): 1 × 10^4^, 2 × 10^4^, and 5 × 10^4^) were respectively co-cultured with A1847-tdTomato^+^ tumor cells (Targets (T): 1 × 10^4^). As expected, all three sources of NK cells were able to efficiently target A1847 cells and lead to immediate apoptosis of tumor cells within 4 h (Fig. 4a, b). Consequently, the A1847 tumor cells were killed sharply after 4-h co-culture with iNK cells at various E:T ratios (Fig. 4c). NK cells can enhance tumor-killing effect by ADCC mechanism^40,41^. To examine the ADCC ability of iNK cells, we combined the anti-CD20 antibody (Rituximab) with iNK cells for killing CD20^+^ human lymphoma cell line Raji at different E:T ratios (1:1, 2:1, 5:1, and 10:1). As expected, the anti-CD20 antibody further enhanced the iNK cell tumor-killing activity against Raji tumor cells (Fig. 4d). Taken together, iNK cells derived from hPSCs possess tumor-killing activity via direct and ADCC manners.

### iNK cells eradicate human tumor cells in xenograft animals

**Fig. 5:**
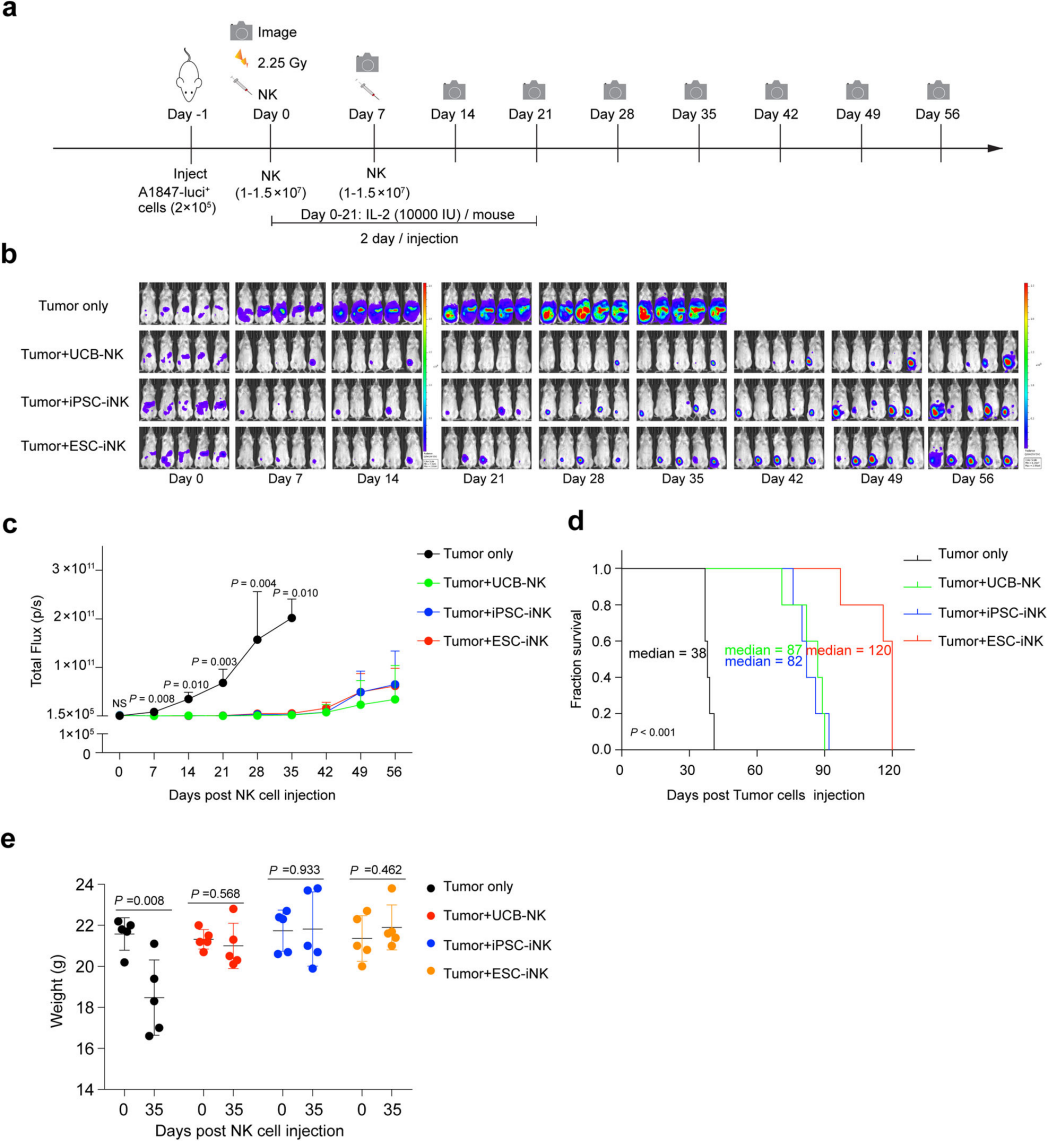
ESC- or iPSC-derived iNK cells eradicate human tumor cells in xenograft animals. **a** Procedure design for NK cell treatment in tumor-bearing animals. **b** BLI images of the xenograft models (Tumor only, Tumor + UCB-NK, Tumor + iPSC-iNK and Tumor + ESC-iNK, *n* = 5 each group). The radiance indicates tumor burden. **c** Statistics of the total flux (p/s) of the xenograft models (Tumor only, Tumor + UCB-NK, Tumor + iPSC-iNK and Tumor + ESC-iNK, *n* = 5 each group). **d** Kaplan-Meier survival curves of the xenograft models (Tumor only, Tumor + UCB-NK, Tumor + iPSC-iNK and Tumor + ESC-iNK, *n* = 5 each group) (*P* < 0.001, Log-rank test). Median survival times were shown. **e** The body weights of treated mice on Day 35 after iNK cell injection. Data were analyzed by one-way ANOVA (**c**) or two-tailed independent *t*-test (**e**). NS, not significant. ESC: human ESC line hPSC-2; iPSC: human iPSC line hPSC-6.

**Fig. 6:**
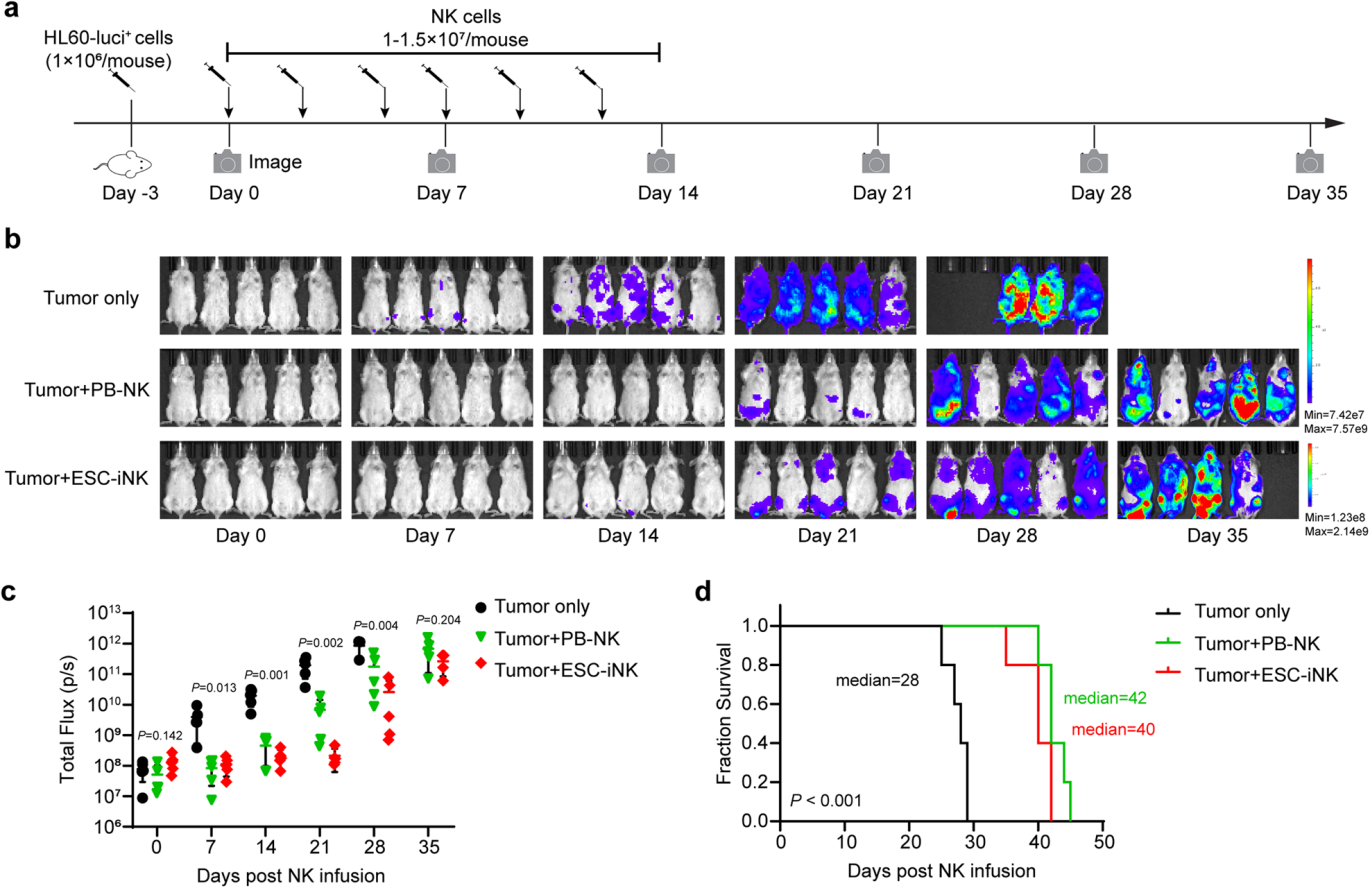
ESC-derived iNK cells inhibit the development of AML in vivo. **a** Diagram of HL-60 cell xenogeneic B-NDG model for ESC-iNK cells against AML target in vivo. Mice were treated intravenously with PB-NK cells or ESC-iNK cells three times a week for 2 weeks. BLI was performed on Day 0, Day 7, Day 14, Day 21, Day 28, and Day 35. **b** Images of individual mouse radiance of the xenograft models (Tumor only, Tumor + PB-NK, and Tumor + ESC-iNK, *n* = 5 each group). The radiance indicates tumor burden. **c** Statistics of the total flux (p/s) of the xenograft models (mean ± SD, *n* = 5). One-way ANOVA and two-tailed independent *t*-test. **d** Kaplan-Meier survival curves of the xenograft models (*P* < 0.001, Log-rank test). Median survival times were shown.

To evaluate the therapeutic potential of the iNK cells in vivo, we established a human tumor xenograft animal model by transplanting the luciferase-expressing A1847 (A1847-luci^+^) cells (2 × 10^5^ cells/mouse) into NCG (a commercial NOD/ShiLtJGpt-*Prkdc*^*em26Cd52*^*Il2rg*^*em26Cd22*^/Gpt strain) mice on Day –1. Then, the iNK cells were intraperitoneally injected into the tumor-bearing animals (1–1.5 × 10^7^ cells/mouse) on Day 0 and Day 7. Meanwhile, UCB-NK cells were used as natural NK cell treatment control. IL-2 (10,000 IU/mouse) was administered every two days until Day 21 to enhance the maintenance of iNK cells in animals^11^. Bioluminescent imaging (BLI) was performed weekly to capture the kinetics of tumor growth (Fig. 5a). Indeed, both ESC-iNK cells and iPSC-iNK cells efficiently eliminated tumor cells in vivo and exhibited comparable tumor-killing efficiencies to natural UCB-NK control group. Meanwhile, the tumor burden of the tumor-only group became increasingly severe, as indicated by the radiance and the value of total flux (Fig. 5b, c), and eventually this group of mice needed ethical euthanasia due to heavy tumor burden between Day 37 and Day 41 post A1847-luci^+^ cell injection. The ESC-iNK cell-treated mice and iPSC-iNK cell-treated mice survived significantly longer than the tumor-only control mice (Tumor only: 38 days; Tumor + UCB-NK: > 87 days; Tumor + iPSC-iNK: > 82 days; Tumor + ESC-iNK: > 120 days; *P* < 0.001) (Fig. 5d). Moreover, the iNK cell therapy treated tumor-bearing animals recovered normal body weights (Fig. 5e). To verify whether iNK cells exhibit anti-tumor activities against different tumor types, we further tested the tumor-killing activity of iNK cells using an acute myeloid leukemia (AML) model. A luciferase-labeled AML cell line (HL60-luci^+^) was intravenously injected into B-NDG hIL15 mice (NOD.CB17-*Prkdc*^*scid*^*Il2rg*^*tm1*^*Il15*^*tm1(IL15)*^/Bcgen background) for the establishment of human AML tumor-bearing animals. Three days after tumor inoculation, mice were treated intravenously with either control human peripheral blood NK (PB-NK) cells or ESC-iNK cells (1–1.5 × 10^7^ iNK cells/mouse) with a procedure of three times a week for continuous 2 weeks (Fig. 6a). The mice were evaluated for tumor burden using bioluminescence every week. In comparison with the nontreatment controls, both human ESC-derived iNK cells and control PB-NK cells inhibited the AML tumor growth significantly (Fig. 6b, c). Moreover, tumor-bearing mice from the ESC-iNK treatment group showed a significantly prolonged median survival (Fig. 6d). Collectively, these results show that the ESC- or iPSC-derived iNK cells can efficiently kill tumor cells and prolong survival of human tumor-bearing animals.

## Discussion

In this study, we develop an EB-free, organoid aggregate method for NK cell regeneration from hPSCs. In a short time-window of 27-day induction, one million hPSC input can output over one billion iNK cells without the necessity of NK cell expansion feeders. Functionally, the iNK cells exhibited strong anti-tumor activity in animal models bearing human tumor cells. At the molecular level, the iNK cells showed the typical expression patterns of activating and inhibitory receptors, cytotoxic molecules, and apoptosis-related ligands of natural NK cell counterparts. Mechanistically, the iNK cells possess promising abilities of killing human tumor cells via typical cytotoxic, ADCC, and apoptotic mechanisms. Our study provides a method for manufacturing large-scale “off-the-shelf” NK cell products for immune therapy.

In general, UCB-NK cells from one individual can expand up to billions of NK cells following two-week expansion with a conventional expansion protocol in the presence of expanding feeder cells^42^. In our study, one batch of hPSCs can produce as many as hundred billion iNK cells with the same genetic background and one single hPSC can produce over 1000 iNK cells in the absence of NK cell expansion feeders. The iNK cell yields per hPSC in our study are much higher than those by the conventional EB induction method (2–12 iNK cells per hPSC on Day 39)^43^. The improvement of efficiency is associated with the generation of pure LPM intermediates and organoid aggregate incubation approach.

We found that the iNK cells exhibited an activated status from as early as newborn, which is different from natural adult NK cells born with naïve status. This phenomenon raises a possibility that the iNK cells possess less functional specification potential than the diversified natural NK cells developed and distributed in multiple organs in humans^44^. In our method, the entire NK induction process only takes four weeks, which is much shorter than the time spent by conventional methods^11,14^. Given the unlimited source of hPSCs, the yields of iNK cells can reach trillions by increasing the input of initiating stem cells. For example, we validated that two million hPSCs can output over 2 × 10^9^ iNK cells with 1-month induction. For further scale-up manufacturing purpose, one billion hPSCs input can produce over one trillion iNK cells, which might meet the application requirements of 100–1000 patients^45^.

We observe that NOTCH ligand DLL1 or DLL4 from microenvironmental feeder cells is dispensable for ESC- and iPSC-derived NK cell induction efficiency, maturation, and cytotoxicity. However, Notch signaling plays a direct, cell-intrinsic role in natural human NK cell terminal maturation^46^. Notch signaling also regulates human NK cell plasticity^47^. One limitation in our current induction approach is that the iNK cells from ESCs or iPSCs are in totally activated forms and we never observed phenotypically resting NK cells, which are naturally developed before activation in humans. Both naturally activated NK cells and induced NK cells show homogenous phenotypes at single cell level and only naturally resting NK cells from human peripheral blood are obviously heterogenous at transcriptome level (Supplementary Fig. S4). Thus, we cannot exclude the possibility that NOTCH ligands might be essential for the heterogenicity of NK cell development since current available induction approaches fail to obtain resting NK cells from ESCs or iPSCs in vitro. In future, once new approach of generating resting NK cells in vitro succeeds, we can further test the roles of NOTCH ligands from feeder cells in terminal maturation and heterogeneity of iNK cells generated from ESCs or iPSCs.

In this study, the iNK cells regenerated from ESCs significantly prolonged survival of A1847 ovarian cancer xenograft models when compared with the groups treated with UCB-NK or iPSC-iNK cells. This phenomenon reappeared even using iNK cells from multiple independent induction assays with ESCs as starting cells. It deserves further investigations whether this is the inherited trait of ESC-derived iNK cells or is caused by different genetic backgrounds of starting cell lines. In future, more hPSC strains with different genetic background need to be broadly assessed. It has been reported that hPSC-derived erythro-myeloid progenitors (EMP)-like NK cells are more cytotoxic than adult NK cells^48^. In this study, we cannot exclude the possibility that the iNK cells from hPSCs in our induction protocol were derived from EMPs.

In addition, we found that the proportion of CD16^+^ iNK cells derived from different stem cells fluctuates with a range from 30% to 90%. However, the rates of CD16^+^ iNK cells from the same stem cell line are highly stable using our approach. Thus, the causing factor of CD16 expression variations is associated with different hPSC lines rather than the induction method itself (Supplementary Fig. S5). This observation also brings an advantage to pre-screen master stem cell lines capable of producing iNK cells with high expression rates of CD16, as CD16^+^ iNK cells are crucial for combinational application of targeting antibodies in treating cancer patients.

## Materials and methods

### Cell culture

Human ESC lines (hPSC-1, hPSC-2, hPSC-3, hPSC-4, and hPSC-5) were provided by National Stem Cell Resource Center, Institute of Zoology, Chinese Academy of Sciences. Human iPSC line (hPSC-6) was derived from a healthy donor’s urine cells by reprogramming, with the donor’s informed consents. All the hPSC lines were maintained in ncTarget medium (Nuwacell) on Matrigel (Corning) coated plates. Peripheral blood samples were obtained from a healthy donor with the donor’s informed consents. Umbilical cord blood samples were obtained from Guangdong Cord Blood Bank (Guangzhou, China). The PB-NK and UCB-NK cells were cultured in the KBM581 medium (Corning) supplemented with IL-2 (200 IU/mL, Miltenyi) and SGR-SM (1%, DAKEWE). OP9 cell line was purchased from ATCC and cultured with α-MEM (Gibco) with 20% fetal bovine serum (FBS) (Ausbian). A1847 cell line was purchased from Honsun Biological Technology Co., Ltd (Shanghai, China) and cultured in RPMI 1640 Medium (Gibco) supplemented with 10% FBS (Ausbian). Luciferase-expressing HL-60 cell line was cultured in RPMI 1640 Medium (Gibco) supplemented with 10% FBS (Ausbian). Raji cell line was purchased from the Cell Resource Center of Shanghai Institutes for Biological Sciences, Chinese Academy of Sciences (Shanghai, China) and cultured in RPMI 1640 Medium (Gibco) supplemented with 10% FBS (Ausbian). K562 cell line was purchased from ATCC and cultured in RPMI 1640 Medium (Gibco) supplemented with 10% FBS (Ausbian).

### Hematopoietic differentiation and NK cell regeneration in vitro

Briefly, for LPM differentiation, the Accutase (400-600 units/mL, Sigma-Aldrich) digested hPSCs were resuspended in the ncTarget medium (Nuwacell) supplemented with thiazovivin (0.5 µM, Selleck), and then were plated on the vitronectin (10 μg/mL) coated dishes. Particularly, LPM differentiation medium was the TeSR™-E6 basal medium (STEMCELL Technologies) supplemented with BMP4 (40 ng/mL, R&D Systems), ACTIVIN A (30 ng/mL, PeproTech), bFGF (20 ng/mL, PeproTech), CHIR99021 (6 µM, Selleck) and PIK-90 (100 nM, Selleck) on Day 0, and supplemented with BMP4 (40 ng/mL, R&D Systems), A-83-01 (1 µM, Selleck) and C59 (1 µM, Selleck) on Day 1. After 24-h induction, LPM cells were digested with Accutase and 0.5 mM EDTA solution (1:1) for 1 min and resuspended in hematopoietic differentiation medium (HDM) composed of TeSR™-E6 medium supplemented with 10 µM SB431542 (selleck), 10 µM Hydrocortisone (selleck), 5 ng/mL Flt3L (PeproTech), 5 ng/mL TPO (PeproTech), 50 ng/mL SCF (PeproTech), 50 ng/mL EGF (PeproTech), 50 ng/mL VEGF (R&D System), 50 ng/mL bFGF (PeproTech), 50 ng/mL IGF-1 (PeproTech), 50 μg/mL ascorbic acid and 2% SGR-SM (DAKEWE). Meanwhile, OP9 or OP9-derived feeder cells expressing human DLL1 or DLL4 were harvested by 0.25% trypsinization (Hyclone) and resuspended in HDM. Then 5 × 10^5^ feeder cells were combined with 2 × 10^4^ LPM cells to form organoid aggregates^22^. Except for induction efficiency, NK cell maturation, and NK cell function comparison purpose, organoid aggregates are prepared by mixing LPM cells with OP9 feeder cells without expressing human NOTCH ligand DLL1 or DLL4. The organoids were plated on a 0.4 µm Millicell transwell insert (Corning) and placed in 6-well plates containing 1 mL HDM per well. The medium was changed completely every 2–3 days for two weeks. On Day 16, the medium was changed to NK differentiation medium^30^. Half medium exchanges were performed every 2 days. On Day 27, the mature NK cells were harvested and cultured in the KBM581 medium (Corning) supplemented with IL-2 (200 IU/mL, Miltenyi) and SGR-SM (1%, DAKEWE).

### Real-time quantitative PCR

The total RNA was extracted by the RNAprep pure Cell Kit (TIANGEN), and 1 µg RNA was reverse-transcribed into cDNA by using ReverTra Ace^®^ qPCR RT Master Mix with gDNA Remover (TOYOBO). Real-time quantitative PCR was performed with Hieff^®^ qPCR SYBR^®^ Green Master Mix (Yeasen Biotechnology). *β-ACTIN* was used for normalization. The relative gene expression level was calculated as 2^–ΔCt^. All primers used in this study were listed in Supplementary Table S1.

### Flow cytometry

Each organoid was incubated in 1 mL digestion buffer (1 mg/mL Collagenase type IV (BasalMedia Technologies), 1 mg/mL Dispase (Gibco) and 50 U DNase I (Sigma-Aldrich)) for 10 min at 37 °C, mechanically disrupted by pipetting, and incubated for another 10 min at 37 °C. After complete disaggregation by pipetting, single cell suspensions were prepared by passage through a 70 µm filter (Corning). Except for organoid, other single cell suspensions were prepared by Accutase and filtered by 70 µm filter. Cells were blocked by Human TruStain FcX™ (Biolegend, 422302) antibody, and then stained with related antibodies. The following antibodies were used: BRACHYURY (R&D System, IC2085A), APLNR (R&D System, FAB8561A-025), CD3 (Biolegend, HIT3a), CD16 (Biolegend, 3G8), CD31 (Biolegend, WM59), CD34 (Biolegend, 581), CD43 (BD Biosciences, 1G10), CD45 (Biolegend, HI30), CD56 (Biolegend, HCD56), CD73 (eBioscience, AD2), CD144 (Biolegend, BV9), CD235a (Biolegend, HI264), DLL4 (Miltenyi, REA1065), NKp30 (Biolegend, P30-15), NKp44 (Biolegend, P44-8), NKG2D (Biolegend, 1D11), CD319 (Biolegend, 162.1), NKG2A (Biolegend, S19004C), CD96 (Biolegend, NK92.39), CD94 (BD Biosciences, HP-3D9), CD69 (Biolegend, FN50), TRAIL (Biolegend, RIK-2), FasL (Biolegend, NOK-1), GzmB (Biolegend, QA18A28), Perforin (Biolegend, dG9). The cells were resuspended in the DAPI (Sigma-Aldrich) solution and were analyzed with BD LSRFortessa X-20 cytometer (BD Biosciences). Flow cytometry data were analyzed by the FlowJo software (Three Star, Ashland, OR, USA).

### RNA-seq and data analysis

The cDNA of single UCB-NK, human ESC-iNK and iPSC-iNK cell sorted on Day 27 was generated and amplified using Discover-sc WTA Kit V2 (Vazyme). The quality of amplified cDNA was assessed by qPCR analysis of *GADPH* gene. Samples that passed quality control were used for sequencing library preparation using TruePrep DNA Library Prep Kit V2 (Vazyme). All libraries were sequenced by Illumina sequencer NextSeq 500. The raw data (fastq files) were generated using bcl2fastq software (version 2.16.10). The raw reads were aligned to human genome hg19 by HISAT2 (version 2.1), and the expression levels in TPM were estimated by StringTie (version 1.3.4). Fifty thousand sorted cells (CD45^+^CD3^–^CD56^+^) from resting PB-NK, activated PB-NK, ESC-iNK and iPSC-iNK populations were used for 10× scRNA-seq. Droplet-based scRNA-seq datasets were produced using a Chromium system (10× Genomics, PN120263) following the manufacturer’s instructions. Droplet-based scRNA-seq datasets were aligned and quantified using the CellRanger software package (version 6.0.0) and subjected to Seurat (version 3.2.3)^49^ for further analysis. Projection of iNK onto PB-NK was performed using the Seurat package. Before integrating data, we performed simple linear regression against the cell cycle score calculated by CellCycleScoring to rule out the effects of cell cycle variances. All datasets were integrated using Seurat’s integration function. Anchors were identified with the FindIntegrationAnchors function, and then the IntegrateData function was used with dim = 1:30. The standard workflow for UMAP dimensionality reduction was performed using the top 30 PCs. All the raw data (fastq files) were uploaded to the Genome Sequence Archive public database (HRA001609).

### IFN-γ and TNF-α staining

iNK cells were incubated with or without PMA/ionomycin (MULTISCIENCES), K562 tumor cells (E:T = 1:2), or recombinant human IL-2 (1000 IU/mL, Miltenyi) for 2 h, followed by adding BFA/Monensin (MULTISCIENCES) for additional 2-h incubation. Cells were stained with anti-human CD45 (Biolegend, HI30), CD3 (Biolegend, HIT3a), and CD56 (Biolegend, HCD56) antibodies. FIX & PERM Kit (MULTISCIENCES) was used for fixation and permeabilization, followed by intracellular staining for IFN-γ (Biolegend, 4 S.B3) or TNF-α (Biolegend, MAb11). The cells were analyzed with BD LSRFortessa X-20 cytometer (BD Biosciences).

### Function analysis of hPSC-derived iNK cells in vitro

iNK cells were derived from hPSCs and UCB-NK cells were isolated from umbilical cord blood. UCB-NK cells were stimulated and expanded on IL-21 Natural Killer Cell Amplification System (Hangzhou Zhongying Biomedical Technology Co., Ltd) for 14 days. ESC-iNK cells, iPSC-iNK cells, and UCB-NK cells (Effector, E) were incubated with 1 × 10^4^ carboxyfluorescein diacetate succinimidyl ester (CFSE; Beijing BioRab Technology Co. Ltd.) labeled A1847, A1847-tdTomato^+^, or K562 cells (Target, T) in flat-bottom 96-well plates for 4 h at respective E:T ratios. The apoptosis of A1847 cells was quantified using Caspase 3/7 Activity Apoptosis Assay Kit (Sangon Biotech). For ADCC analysis, ESC-iNK cells, iPSC-iNK cells and UCB-NK cells (Effector, E) were incubated with 1 × 10^4^ CFSE-labeled Raji cells (Target, T) by adding anti-CD20 antibody (Rituximab, 20 μg/mL, Selleck) in U-bottom 96-well plates for 4 h at respective E:T ratios (E:T = 0:1, 1:1, 2:1, 5:1, 10:1). Target cell death was assessed with flow cytometer (BD LSRFortessa X-20 cytometer, BD Biosciences) by the percentage of DAPI in CFSE-positive population. Flow cytometry data were analyzed by the FlowJo software (Three Star, Ashland, OR, USA).

### Construction of the ovarian cancer xenograft models and treatment with iNK cells

NCG mice (NOD/ShiLtJGpt-*Prkdc*^*em26Cd52*^*Il2rg*^*em26Cd22*^/Gpt, GemPharmatech Co., Ltd.) were intraperitoneally injected with the luciferase-expressing A1847 (A1847-luci^+^) cells (2 × 10^5^ cells/mouse) to construct the ovarian cancer xenograft models on Day –1. BLI (IVIS Spectrum PerkinElmer) was performed on these models to quantify the tumor burden, and the models with similar total flux (p/s) were randomly divided into four groups (Tumor only, Tumor + UCB-NK, Tumor + iPSC-iNK and Tumor + ESC-iNK) on Day 0. These models were first irradiated (2.25 Gy, Rad Source RS2000), and then intraperitoneally injected with the ESC-iNK, iPSC-iNK, or UCB-NK cells (1–1.5 × 10^7^ cells/mouse) 4 h post irradiation and 7 days after the first injection. IL-2 (10,000 IU/mouse) was administered every two days until Day 21 post NK cell injection. BLI was performed every week to trace the tumor cells. Mice suffering from heavy tumor burden were euthanized for ethical consideration.

### Construction of the AML xenograft models and iNK cell treatment

B-NDG hIL15 mice (NOD.CB17-*Prkdc*^*scid*^*Il2rg*^*tm1*^*Il15*^*tm1(IL15)*^/Bcgen background, Biocytogen Co., Ltd) were intravenously injected with the luciferase-expressing HL-60 (HL60-luci^+^) cells (1 × 10^6^ cells/mouse) to construct the AML xenograft models on Day –3. Three days later, BLI (IVIS Spectrum PerkinElmer) was performed on these models to quantify the tumor burden, and the models with similar total flux (p/s) were randomly divided into three groups (Tumor only, Tumor + PB-NK, and Tumor + ESC-iNK). Then, these models were intravenously injected with the PB-NK or ESC-iNK three times a week for 2 weeks. Tumor burden was assessed every week from Day 0 using BLI. Mice suffering from heavy tumor burden were euthanized for ethical consideration.

### Ethical statement

NCG mice and B-NDG hIL15 mice were housed in the SPF-grade animal facility of the Guangzhou Institutes of Biomedicine and Health, Chinese Academy of Sciences and the Institute of Zoology, Chinese Academy of Sciences. All procedures of this study were approved by the Institutional Animal Care and Use Committee of the Institute of Zoology, and the Institutional Animal Care and Use Committee of the Guangzhou Institutes of Biomedicine and Health. hPSC differentiation toward hematopoietic lineage cells and immune cells, and the related anti-tumor activity assessments of iNK cells in animals are approved by the Biomedical Research Ethics Committee of the Institute of Zoology, Chinese Academy of Sciences. The iPSC preparations from donor somatic cells and human peripheral blood in this study are also permitted with donor consent.

### Statistics

All quantitative analyses were performed with SPSS (SPSS v.23, IBM Corp., Armonk, NY, USA). Normal distribution of data was tested with SPSS applying Shapiro-Wilk normality test. Two-tailed independent *t*-test and Mann-Whitney *U* test were performed for comparison of two groups of data. For three groups or more, one-way ANOVA and Kruskal-Wallis tests were used. Kaplan-Meier method was used to plot survival curves of leukemia, and Log-rank (Mantel-Cox) test was performed to compare differential significance in survival rates.

## Supplementary information

Supplementary information
