# Author Correction: Lateral plate mesoderm cell-based organoid system for NK cell regeneration from human pluripotent stem cells

**DOI:** 10.1038/s41421-023-00526-2

**Published:** 2023-02-21

**Authors:** Dehao Huang, Jianhuan Li, Fangxiao Hu, Chengxiang Xia, Qitong Weng, Tongjie Wang, Huan Peng, Bingyan Wu, Hongling Wu, Jiapin Xiong, Yunqing Lin, Yao Wang, Qi Zhang, Xiaofei Liu, Lijuan Liu, Xiujuan Zheng, Yang Geng, Xin Du, Xiaofan Zhu, Lei Wang, Jie Hao, Jinyong Wang

**Affiliations:** 1 State Key Laboratory of Stem Cell and Reproductive Biology, Institute for Stem Cell and Regeneration, Institute of Zoology, Chinese Academy of Sciences, Beijing, China; 2 CAS Key Laboratory of Regenerative Biology, Guangzhou Institutes of Biomedicine and Health, Chinese Academy of Sciences, Guangzhou, Guangdong China; 3 University of Chinese Academy of Sciences, Beijing, China; 4 Beijing Institute for Stem Cell and Regenerative Medicine, Beijing, China; 5 Department of Hematology, Guangdong General Hospital, Guangzhou, Guangdong China; 6 Department of Pediatrics, State Key Laboratory of Experimental Hematology, National Clinical Research Center for Blood Diseases, Institute of Hematology & Blood Diseases Hospital, Chinese Academy of Medical Sciences & Peking Union Medical College, Tianjin, China; 7 National Stem Cell Resource Center, Chinese Academy of Sciences, Beijing, China

**Keywords:** Stem-cell differentiation, Tumour immunology

Correction to: *Cell Discovery* (2022) 8:121

10.1038/s41421-022-00467-2 published online 08 November 2022

In the original publication of this article, the authors have found an error in Acknowledgements section, the fund of 2021YFA1100701 should not be included. Now we provided a corrected version here.

