# Supplementary material for: Lateral plate mesoderm cell-based organoid system for NK cell regeneration from human pluripotent stem cells
Source: Cell Discov. 2022 Nov 8;8:121. doi: 10.1038/s41421-022-00467-2 (PMC9640545; doi:10.1038/s41421-022-00467-2)
Supplement: Supplementary file 1 — Supplementary information [file 41421_2022_467_MOESM1_ESM.pdf]

Supplementary information

Lateral plate mesoderm cell-based organoid system for NK cell regeneration from human pluripotent stem cells

Dehao Huang[1#], Jianhuan Li[2,3,#], Fangxiao Hu[4#], Chengxiang Xia[4#], Qitong Weng[1], Tongjie Wang[1], Huan Peng[2,3], Bingyan Wu[2,3], Hongling Wu[2], Jiapin Xiong[2,3], Yunqing Lin[1,3], Yao Wang[2,3], Qi Zhang[1], Xiaofei Liu[2], Lijuan Liu[1], Xiujuan Zheng[2,3], Yang Geng[2], Xin Du[5], Xiaofan Zhu[6], Lei Wang[1,7], Jie Hao[1,7], Jinyong Wang[1,3,4,*]

[1]State Key Laboratory of Stem Cell and Reproductive Biology, Institute for Stem Cell and Regeneration, Institute of Zoology, Chinese Academy of Sciences, Beijing 100101, China.

[2]CAS Key Laboratory of Regenerative Biology, Guangzhou Institutes of Biomedicine and Health, Chinese Academy of Sciences, Guangzhou 510530, China.

[3]University of Chinese Academy of Sciences, Beijing 100049, China.

[4]Beijing Institute for Stem Cell and Regenerative Medicine, Beijing 100101, China.

[5]Department of Hematology, Guangdong General Hospital, Guangzhou 510080, China.

[6]Department of Pediatrics, State Key Laboratory of Experimental Hematology, National Clinical Research Center for Blood Diseases, Institute of Hematology & Blood Diseases Hospital, Chinese Academy of Medical Sciences & Peking Union Medical College, Tianjin 300020, China

[7]National Stem Cell Resource Center, Chinese Academy of Sciences, Beijing 100190, China.

These authors contributed equally: Dehao Huang, Jianhuan Li, Fangxiao Hu, Chengxiang Xia

*Correspondence: Jinyong Wang (wangjinyong@ioz.ac.cn)

Supplementary Figures and Figure legends

Supplementary Fig.S1

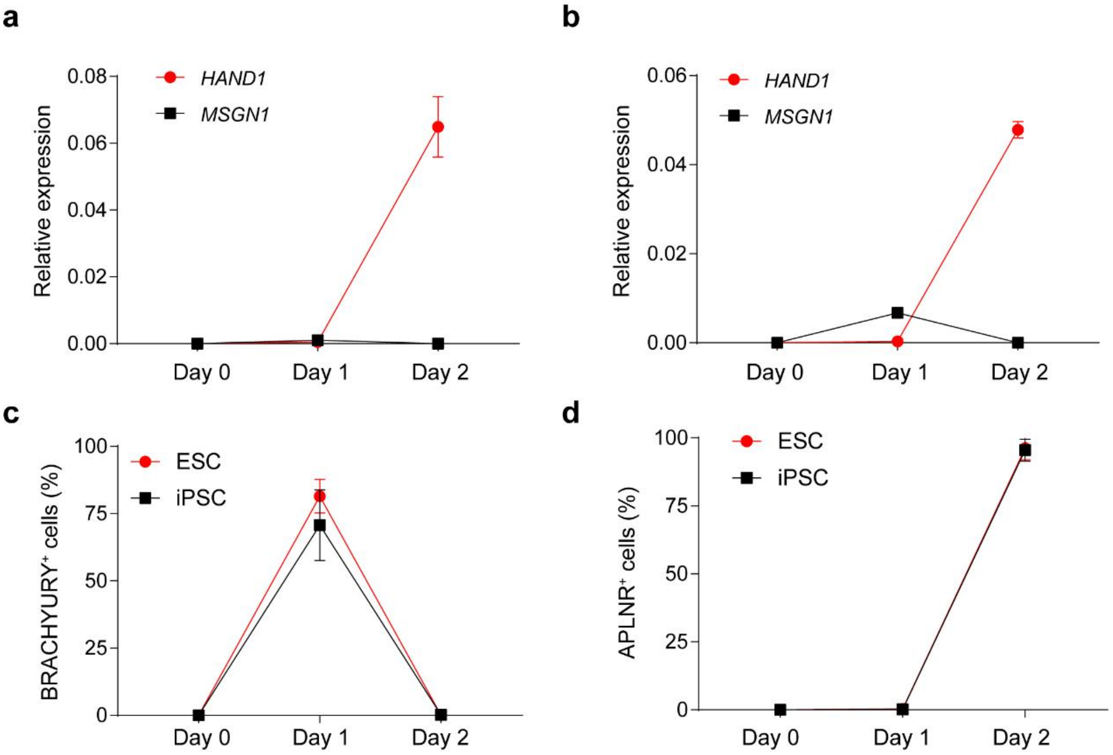

Supplementary Fig. S1 Analysis of primitive streak and lateral plate mesoderm-specific markers of hPSC-derived cells

a Real-time quantitative PCR analysis of *HAND1* and *MSGN1* gene expression levels of ESC-derived cells on Day 0, Day 1, and Day 2. **b** Real-time quantitative PCR analysis of *HAND1* and *MSGN1* gene expression levels of iPSC-derived cells on Day 0, Day 1, and Day 2. **c** The proportion of $BRACHYURY^+$ cells in hPSC-derived cells on Day 0, Day 1, and Day 2. Data were collected from three independent experiments. $n = 3$. **d** The proportion of $APLNR^+$ cells in hPSC-derived cells on Day 0, Day 1, and Day 2. Data were collected from three independent experiments. $n = 3$. ESC: human ESC line hPSC-2; iPSC: human iPSC line hPSC-6.

Supplementary Fig.S2

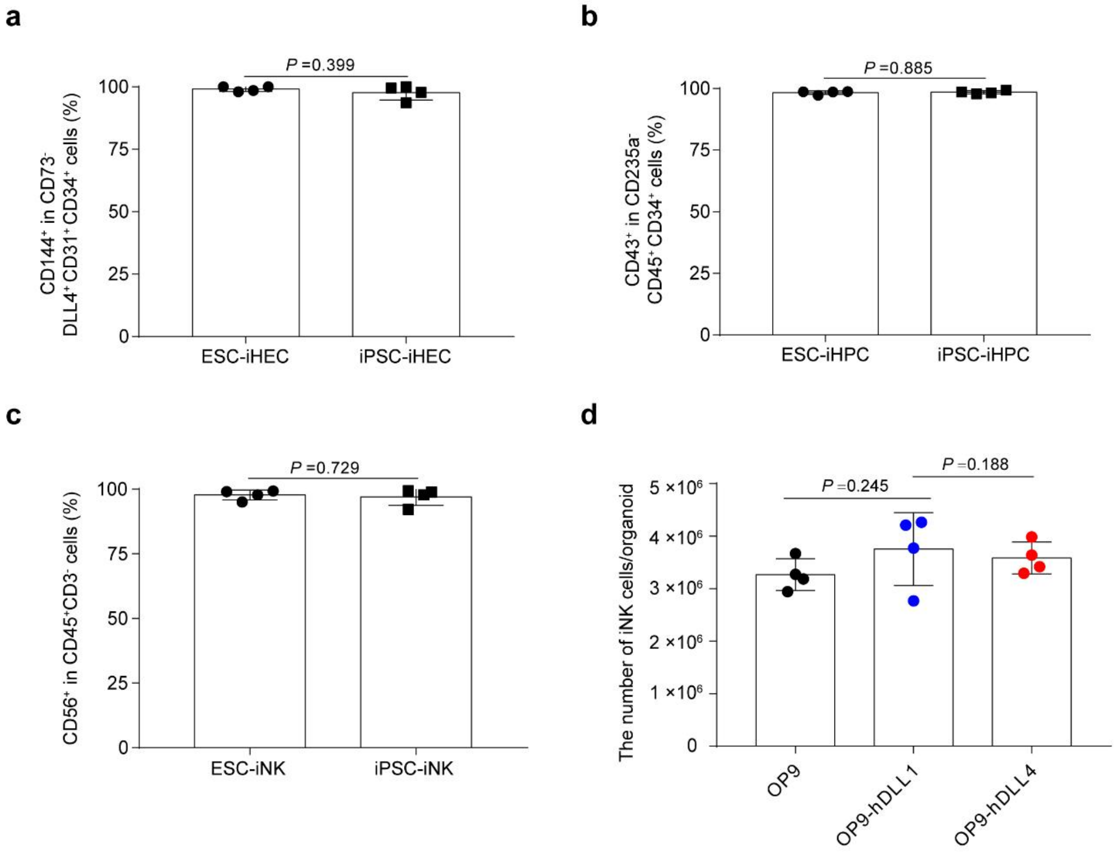

Supplementary Fig. S2 Analysis of the efficiency of iHEC, iHPC, and iNK cell regeneration from hPSCs

a The proportion of CD144$^+$ cells in CD73$^-$DLL4$^+$CD31$^+$CD34$^+$ iHECs on Day 9. Data were collected from four independent experiments and analyzed by two-tailed independent *t*-test. n = 4. **b** The proportion of CD43$^+$ cells in CD235a$^-$CD45$^+$CD34$^+$ iHPCs on Day 16. Data were collected from four independent experiments and analyzed by Mann-Whitney U test. n = 4. **c** The proportion of CD56$^+$ cells in CD45$^+$CD3$^-$ cells on Day 27. Data were collected from four independent experiments and analyzed by two-tailed independent *t*-test. n = 4. **d** 5×10^5 OP9, OP9-hDLL1, and OP9-hDLL4 feeder cells were combined with 2×10^4 mesoderm cells separately to form organoid aggregates on Day 2. Data were collected and analyzed on Day 27 (n = 4 each group). Statistics: two-tailed independent *t*-test.

Supplementary Fig.S3

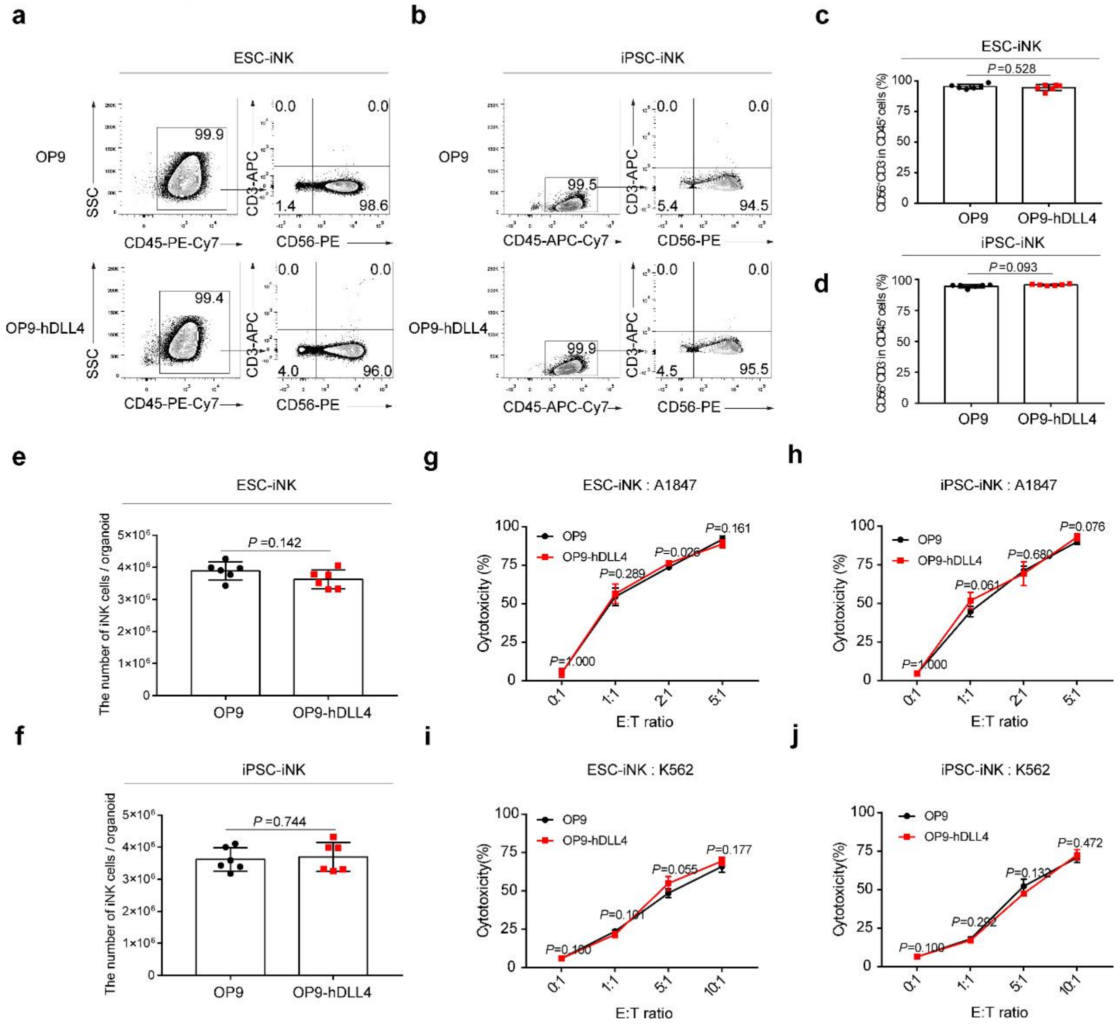

Supplementary Fig. S3 Comparison of the induction efficiency, maturation, and cytotoxicities of iNK cells from ESCs and iPSCs using OP9 and OP9-hDLL4 feeder cells.

a-b Immuno-phenotypes (CD45$^+$CD3$^-$CD56$^+$) of iNK cells from ESC (**a**) and iPSCs (**b**) on Day 27. **c-d** Statistics of the maturation of iNK cells from ESC (**c**) and iPSCs (**d**) on Day 27 (n = 6 each group). **e-f** Statistics of the number of CD45$^+$CD3$^-$CD56$^+$ iNK cells on Day 27 (n = 6 each group). **g-h** Cytotoxicity analysis of ESC-iNK cells (**g**) and iPSC-iNK cells (**h**) against A1847 cell line (n = 4 each group). **i-j** Cytotoxicity analysis of ESC-iNK cells (**i**) and iPSC-iNK cells (**j**) against k562 cell line (n = 4 each group). Statistics: two-tailed independent t-test (**c-j**) and Mann-Whitney U test (**g**).

Supplementary Fig.S4

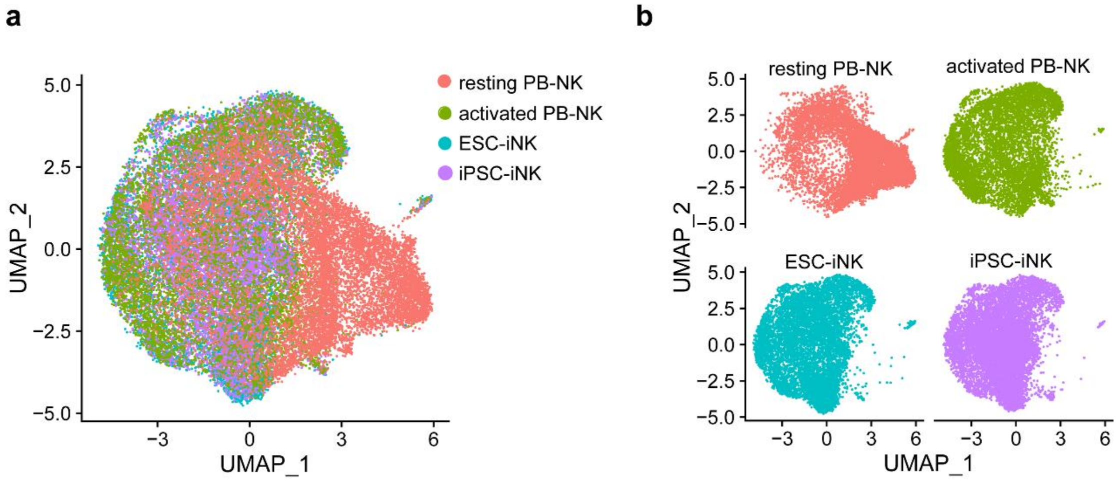

Supplementary Fig. S4 Integration of scRNA-seq data generated from ESC-iNK, iPSC-iNK, resting PB-NK, and activated PB-NK cells.

a Projection of ESC-iNK, iPSC-iNK, resting PB-NK, and activated PB-NK cells. **b** UMAP visualization of ESC-iNK, iPSC-iNK, resting PB-NK, and activated PB-NK cells respectively.

Supplementary Fig.S5

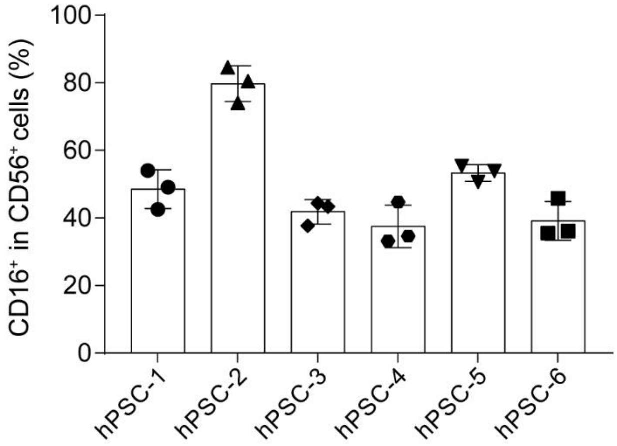

Supplementary Fig. S5 CD16 molecule expression pattern of iNK cells.

CD16 expression levels of iNK cells and NK cells from human embryonic cell line hPSC-1, hPSC-2, hPSC-3, hPSC-4, hPSC-5, and human iPSC line hPSC-6. $CD16^+$ cells were gated on $CD45^+CD3^-CD56^+$ iNK cells. Data were collected from three independent experiments. All samples were collected on Day 27.

Supplementary Table S1

Primer sequences for real-time quantitative PCR of selected genes	
Primer name	Primer sequence (5’→3’)
β-ACTIN-F	CATGTACGTTGCTATCCAGGC
β-ACTIN-R	CTCCTTAATGTCACGCACGAT
TBXT-F	TATGAGCCTCGAATCCACATAGT
TBXT-R	CCTCGTTCTGATAAGCAGTCAC
APLNR-F	CTCTGGACCGTGTTTCGGAG
APLNR-R	GGTACGTGTAGGTAGCCCACA
HAND1-F	GAGAGCATTAACAGCGCATTCG
HAND1-R	CGCAGAGTCTTGATCTTGGAGAG
MSGN1-F	AACCTGCGCGAGACTTTCC
MSGN1-R	GTCTGTGAGTTCCCCGATGTA
